# Isolation, Identification and Antifungal Activity Determination of Antagonistic Bacteria Against *Pyricularia oryzae* Causing Foxtail Millet Blast

**DOI:** 10.3390/plants15213289

**Published:** 2026-10-25

**Authors:** Peixue Xuan, Jia Liu, Mengya Zhang, Jifang Ma, Qian Liu, Hui Bai, Zhiyong Li

**Affiliations:** 1 Institute of Millet Crops, Hebei Academy of Agriculture and Forestry Sciences, Shijiazhuang 050035, China; xpx1996@163.com (P.X.);; 2 Key Laboratory of Genetic Improvement and Utilization for Featured Coarse Cereals (Co-Construction by Ministry and Province), Ministry of Agriculture and Rural Affairs, Institute of Millet Crops, Hebei Academy of Agriculture and Forestry Sciences, Shijiazhuang 050035, China; 3 Key Laboratory of Minor Cereal Crops of Hebei Province, Institute of Millet Crops, Hebei Academy of Agriculture and Forestry Sciences, Shijiazhuang 050035, China; 4 School of Landscape and Ecological Engineering, Hebei University of Engineering, Handan 056038, China

**Keywords:** *Pyricularia oryzae*, foxtail millet, *Pseudomonas silesiensis*, antagonism, biological control

## Abstract

Foxtail millet blast, caused by *Pyricularia oryzae*, is a widespread airborne disease that severely threatens the sustainable production and industrial development of foxtail millet. Developing eco-friendly and non-toxic management strategies has been widely acknowledged as a core prerequisite for establishing sustainable disease control systems. In this study, a bacterial strain NR2065B9 with prominent *in vitro* antagonistic activity against *P. oryzae* was isolated from healthy foxtail millet rhizosphere soil. Taxonomic identification via morphological characterization and 16S rRNA gene sequence homology analysis confirmed that this strain belonged to the species *Pseudomonas silesiensis*. Dual culture assays revealed that strain NR2065B9 exerted a significant inhibitory effect on the mycelial growth of *P. oryzae*. Detached-leaf inoculation assays demonstrated that strain NR2065B9 could remarkably restrict the expansion of blast lesions on foxtail millet leaf tissues. Field trials confirmed that NR2065B9 application could reduce foxtail millet blast severity compared with the controls. Collectively, these findings indicated that the antagonistic strain NR2065B9 possessed great application potential in the biocontrol of foxtail millet blast, and could be further exploited as a promising microbial resource for the development of biocontrol agents against this disease.

## 1. Introduction

Foxtail millet (*Setaria italica* L.), a nutrient-dense cereal crop with high nutritional value, is recognized as one of the most important grain crops in China [1]. In recent years, the extensive adoption of large-scale continuous cropping, single cropping structure of variety, and widespread inherent susceptibility of dominant production varieties to phytopathogens have collectively exacerbated the severity of disease outbreaks in foxtail millet production systems [2].

The causal agent of foxtail millet blast is *Pyricularia oryzae*, whose teleomorph is *Magnaporthe oryzae*. It is an ascomycete belonging to the order Magnaporthales and family Magnaporthaceae/Pyriculariaceae. *P. oryzae* is the most destructive member of the genus *Pyricularia*, and together with its related species *P. grisea*, constitutes the most extensively studied species pair among the blast fungi of gramineous hosts. The two species are closely related and morphologically similar. Because no reliable morphological characters separate them, their names were long treated as synonymous, and they were resolved as distinct cryptic species only through multilocus phylogenetic analysis, which assigned isolates from rice and other grasses to *P. oryzae* and those from crabgrass to *P. grisea*. This close relationship is further reflected in their overlapping host and tissue ranges: certain *P. oryzae* isolates are cross-infective between rice and green bristlegrass, indicating that host shifts may occur between *P. oryzae* and *P. grisea* [3]. At the tissue level, both species are able to invade underground as well as aerial organs; their infection hyphae penetrate intercellular spaces and extend into root cells irrespective of pathotype or host plant [4]. *P. oryzae* itself can infect multiple gramineous crops including foxtail millet, rice, wheat, barley and finger millet, or weeds including goose grass, cutgrass and switchgrass [5]. *P. oryzae* can infect almost all parts of the host plant, forming typical spindle-shaped necrotic lesions with gray centers and dark brown borders on leaves, leaf collars, necks and panicles [6], which often causes severe yield loss in foxtail millet production areas [7]. This pathogen is capable of invading foxtail millet plants across all developmental stages, from early seedling emergence to the late grain-filling phase, with leaf blast and panicle blast representing the two most prevalent damaging disease manifestations [8]. This disease typically causes yield losses exceeding 30%, while in severe epidemic scenarios it can trigger large-scale grain yield reduction or even total crop failure. For highly susceptible cultivars, the recorded yield loss can reach 80% or higher [9]. Currently, foxtail millet blast has become a widespread endemic disease across all major foxtail millet production regions in China, posing a persistent and severe threat to the sustainable and healthy development of this cereal industry [10].

To date, the primary management strategies deployed for foxtail millet blast control is synthetic fungicide application [11]. Chemical control offers the advantages of rapid disease suppression and convenient field implementation, but long-term intensive application of synthetic fungicides can drive the evolution of fungicide resistance in pathogen populations. Furthermore, the associated pesticide residues pose potential risks to food safety, agroecosystem balance, and human health, raising growing public and regulatory concerns [12]. Against this background, the adoption of biological control strategies, particularly the exploration and deployment of antagonistic biocontrol microorganisms, has emerged as a promising and critical alternative for the sustainable, green management of foxtail millet blast.

Rhizosphere-associated bacteria represent the most dominant functional population in plant rhizosphere microecosystems, constituting an enormous resource pool for biocontrol agent discovery [13]. A range of rhizobacteria can mediate multiple beneficial effects including plant growth promotion, induced systemic resistance, and stress tolerance via diverse direct and indirect mechanisms, and have been widely validated for practical applications in agricultural production and sustainable disease management [14]. A previous study has consistently demonstrated that rhizosphere-derived biocontrol bacteria not only enhance plant defensive capacity against phytopathogens, but also exert significant growth-promoting effects on plant vegetative and reproductive development [15]. Accordingly, the targeted isolation and characterization of antagonistic bacteria from native foxtail millet rhizosphere habitats carries important theoretical significance and substantial practical value for the sustainable biocontrol of foxtail millet blast.

To address this urgent need, the present study will isolate culturable functional bacteria with robust antagonistic activity against foxtail millet blast pathogen *P. oryzae* from healthy foxtail millet rhizosphere soil. Strains with high biocontrol potential will be subsequently screened via dual culture assays, and the taxonomic identity of the target antagonistic isolate will be confirmed using a polyphasic approach integrating morphological characterization and 16S rRNA gene sequence phylogenetic analysis. Furthermore, the suppressive efficacy of the selected antagonistic bacterium against foxtail millet blast pathogen will be validated through a combined assay system consisting of *in vitro* detached-leaf inoculation bioassays and field trials. The findings generated from this study will not only advance the fundamental understanding of this functional strain as an effective biocontrol agent, but also provide a promising candidate microbial resource to support the sustainable, eco-friendly development of the foxtail millet industry.

## 2. Results

### 2.1. The Growth Characteristics of Foxtail Millet Blast Pathogen

**Figure 1:**
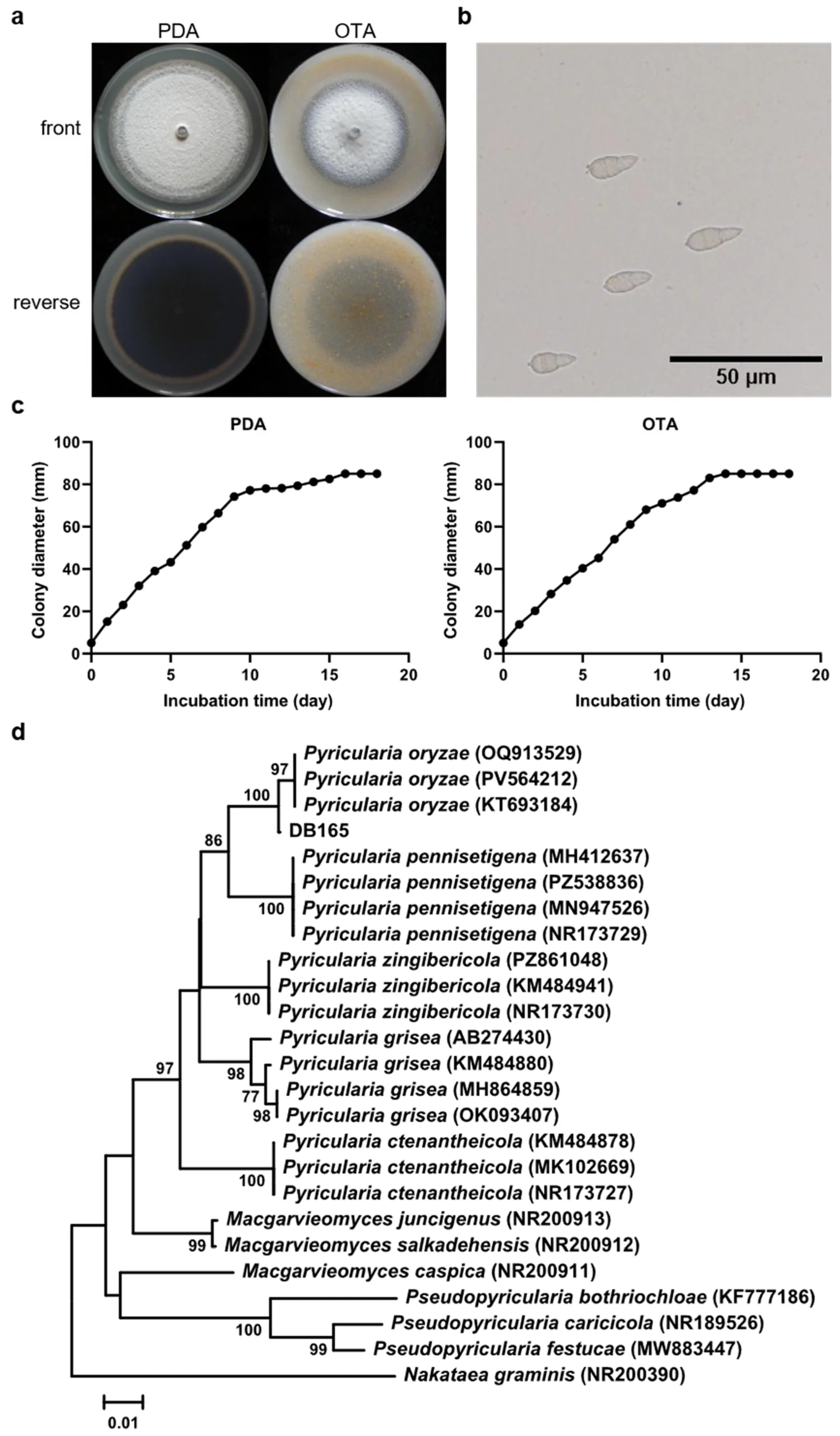
Growth characteristics and molecular identification of *Pyricularia oryzae* DB165. (**a**) Colony morphology on PDA and OTA media. (**b**) Spore morphology under 200× magnification (scale bar = 50 μm). (**c**) Colony diameter on PDA and OTA media (*n* = 5). (**d**) Phylogenetic tree based on rDNA ITS sequence. Distances and clustering were performed using the Neighbor-Joining method. Sequence accession numbers of individual member strains are in parentheses. Numbers at branches indicate bootstrap values (percentages of 1000 replicates). Scale bar represents 0.01 substitutions per site. *Nakataea graminis* was used as the outgroup.

A single-spore purified strain DB165 was isolated from typical blast lesions collected from the experimental station, and this strain was used as the target pathogen for all subsequent experiments in this study. Observation of the colony morphology showed that on potato dextrose agar (PDA) medium, the strain DB165 had dense hyphae, well-developed aerial hyphae, and flocculent morphology. The colony initially appeared white, later became gray, and had white edges. Strain DB165 had sparse aerial hyphae and no secretions on oat tomato agar (OTA) medium. The inner zone of the colony was white, while the outer periphery was gray (Figure 1a). Under the optical microscope, the shape of the conidia was typically pyriform with a rounded base, narrow apex, and 2 septa (Figure 1b). The size of the conidia ranged from 11.86 μm to 18.43 μm in length and 3.95 μm to 6.59 μm in width (*n* = 20, Supplementary Table S1). The growth measurement results showed that the colony required a 18-day continuous incubation period to achieve full surface coverage on PDA medium, with an average growth rate of 6.07 mm/d (Figure 1c, Supplementary Table S2). Complete colonization of the entire OTA medium plate was accomplished within 18 days post-inoculation, with an average growth rate of 5.31 mm/d (Figure 1c, Supplementary Table S3).

The rDNA ITS sequence of DB165 contained 543 base pairs (bp). Preliminary comparisons with rDNA ITS sequence deposited in GenBank coupled with phylogenetic tree analysis indicated that DB165 was most closely related to *P. oryzae* (KT693184), with 533 identical bases out of the total 537 aligned fragment, corresponding to a 99.26% sequence similarity (Supplementary Table S4). The phylogenetic analysis results showed that strain DB165 had the closest phylogenetic relationship with *P. oryzae* (KT693184), with a branch support value of 100% (Figure 1d). Combining morphological and molecular phylogenetic analysis, the foxtail millet blast pathogen DB165 was identified as *P. oryzae*.

### 2.2. Isolation and Identification of Antagonistic Bacteria Against Foxtail Millet Blast Pathogen

**Figure 2:**
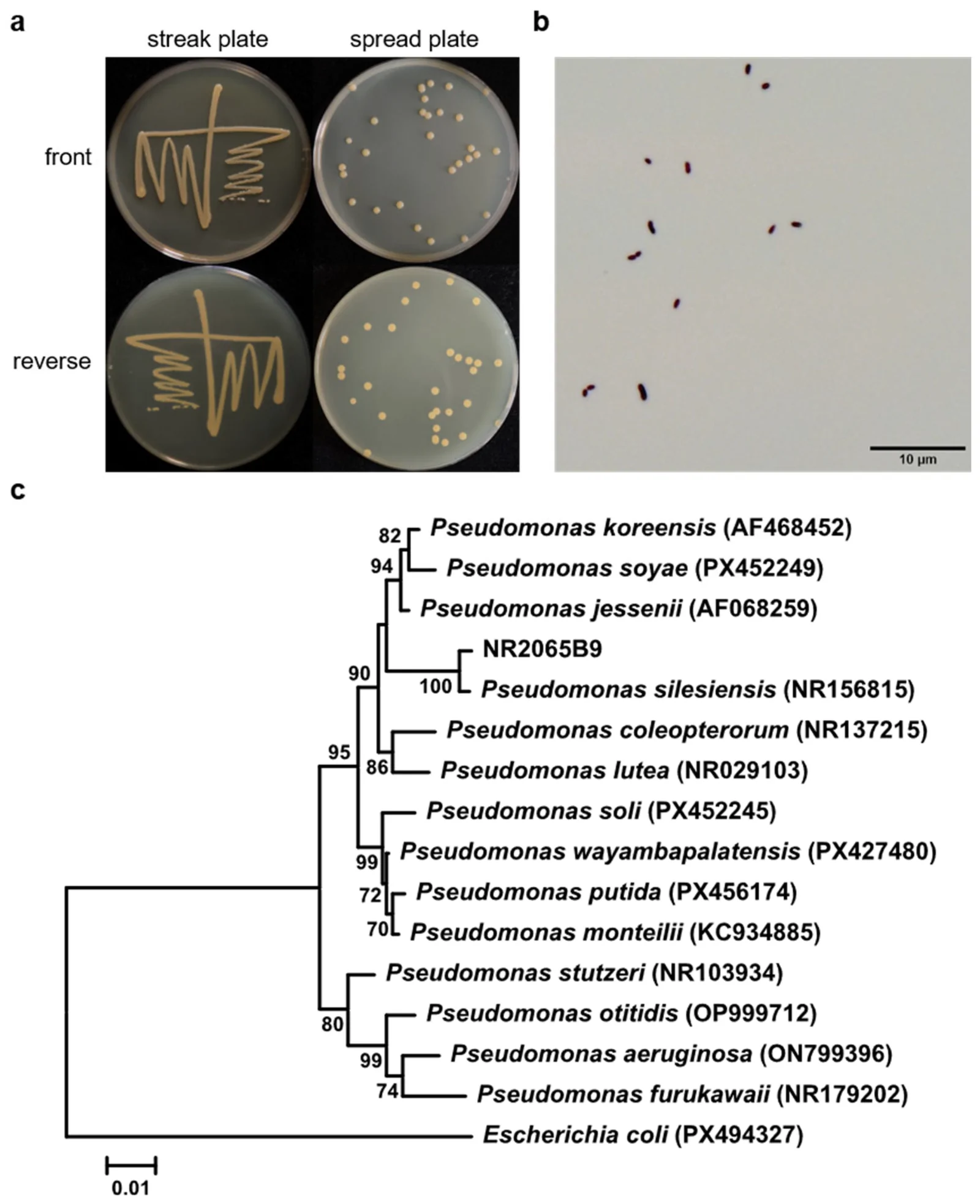
Morphological characteristics and molecular identification of *Pseudomonas silesiensis* NR2065B9. (**a**) Colony morphology on LB solid media. (**b**) The morphology of bacterial cells after Gram staining under 1000× magnification (scale bar = 10 μm). (**c**) Phylogenetic tree based on 16S rRNA gene sequence. Distances and clustering were performed using the Neighbor-Joining method. Sequence accession numbers of individual member strains are in parentheses. Numbers at branches indicate bootstrap values (percentages of 1000 replicates). Scale bar represents 0.01 substitutions per site. *Escherichia coli* was used as the outgroup.

The bacterial strain was isolated and screened from collected soil samples using the plate dilution coating method, with *P. oryzae* set as the target indicator pathogen. The dual culture assay was adopted as the screening approach to select isolates with significant antifungal activity against the pathogen. After *in vitro* antagonism screening, a total of nine isolates with significant inhibitory effects against the foxtail millet blast pathogen *P. oryzae* were obtained (Supplementary Table S5). The bacterial strain NR2065B9, which exhibited the strongest inhibitory activity, was selected for the subsequent experiments. We observed the colony morphology of the isolated strain NR2065B9, and the results showed that after 3 days of cultivation on Luria–Bertani (LB) solid medium at 28 °C, the single colony of strain NR2065B9 appeared yellow, round, with intact edges, raised, and a smooth and moist surface (Figure 2a). Under the optical microscope, it was observed that the bacterial morphology of strain NR2065B9 was straight rod-shaped, and after Gram staining, it exhibited a red color and was identified as Gram-negative bacterium (Figure 2b). The size of the cells ranged from 0.7 μm to 1.69 μm in length and 0.23 μm to 0.52 μm in width (*n* = 20, Supplementary Table S6).

The 16S rRNA gene sequence of the isolate NR2065B9 contained 1440 bp. Preliminary comparisons with 16S rRNA gene sequence deposited in GenBank coupled with phylogenetic tree analysis indicated that NR2065B9 was most closely related to *P. silesiensis* (NR156815), with 1409 identical base pairs out of the total 1417 aligned fragment, corresponding to a 99.44% sequence similarity. The phylogenetic analysis results showed that strain NR2065B9 had the closest phylogenetic relationship with *P. silesiensis* (NR156815), with a bootstrap value of 100% (Figure 2c). Based on morphological and molecular phylogenetic analysis, the isolated bacterium NR2065B9 was identified as *P. silesiensis*.

### 2.3. In Vitro Antagonistic Activity of Functional Bacteria NR2065B9

**Figure 3:**
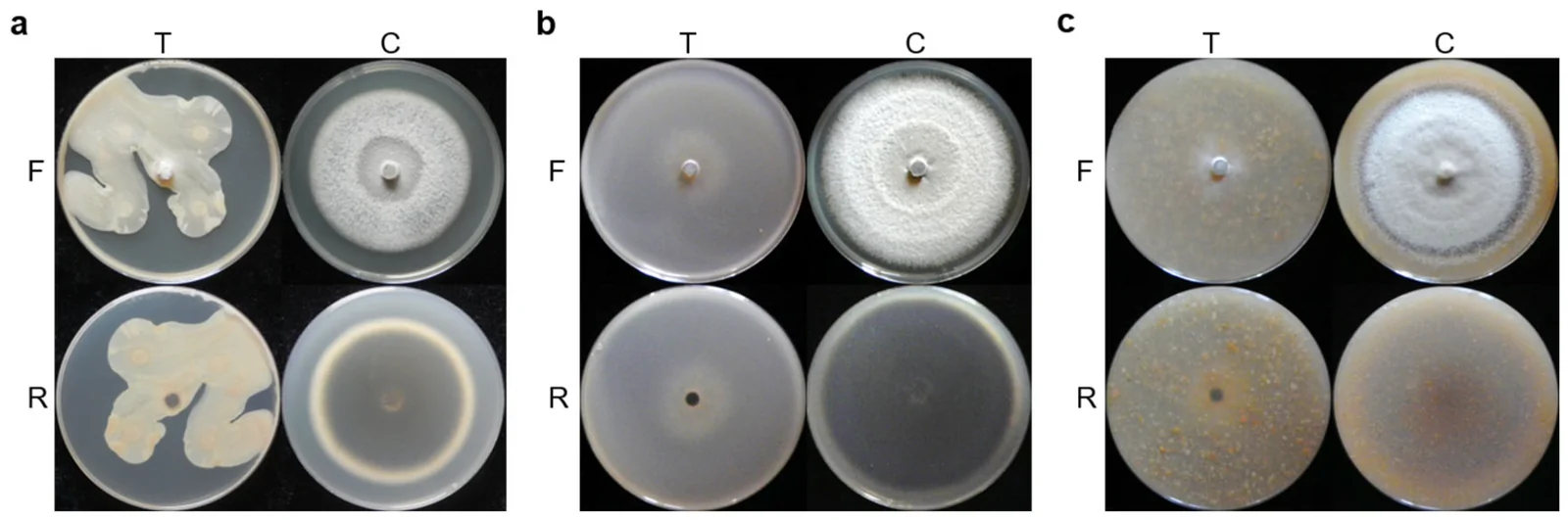
*In vitro* antagonistic effects of *Pseudomonas silesiensis* NR2065B9 on mycelial growth of the foxtail millet blast pathogen DB165 across different culture media. (**a**) Dual culture assay on PDA media. (**b**) PDA media. (**c**) OTA media. T: treatment; C: control; F: front; R: reverse.

The dual culture assay on the PDA medium exhibited a great inhibition of mycelial growth of *P. oryzae* DB165 (Figure 3a), with an inhibition rate of 98.77% (Supplementary Table S7), while growth inhibition was not observed in the non-treated control.

When the foxtail millet blast pathogen DB165 was inoculated onto PDA medium amended with the antagonistic strain NR2065B9, its mycelial growth was significantly inhibited, with colony expansion severely restricted and nearly confined to the vicinity of the inoculation site (Figure 3b,c). The inhibition rate of NR2065B9 was 83.97% on PDA medium and 91.05% on OTA medium (Supplementary Table S8). In the untreated control group, the phytopathogenic fungus exhibited vigorous vegetative hyphal growth, characterized by unconstrained omnidirectional radial expansion across the agar media.

### 2.4. Disease Control of NR2065B9 Suspension Against Foxtail Millet Blast Under Indoor Conditions

**Figure 4:**
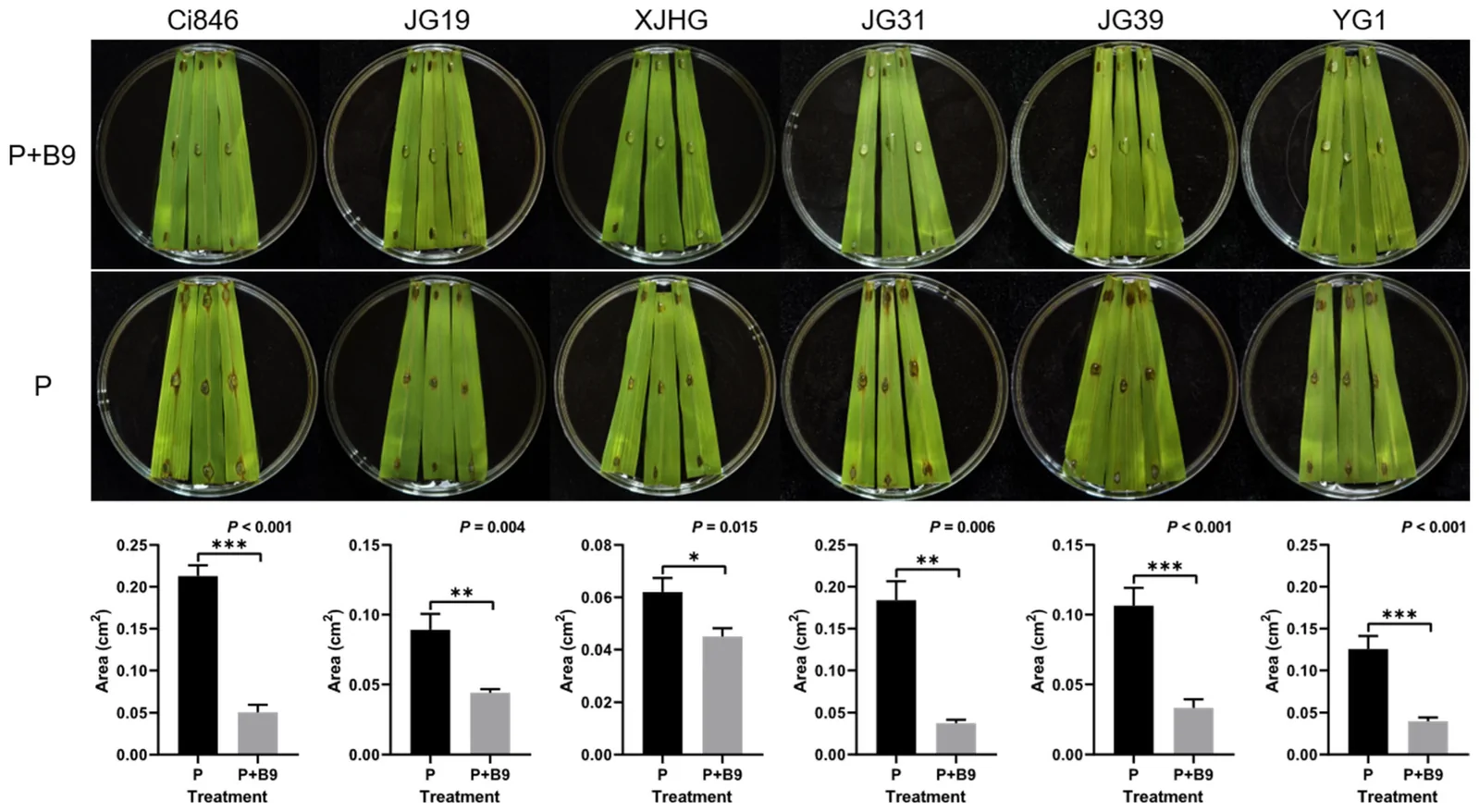
Measurement of foxtail millet blast lesion expansion on leaves under treatments with or without antagonistic bacterium *Pseudomonas silesiensis* NR2065B9. * indicates *p* < 0.05, ** indicates *p* < 0.01, *** indicates *p* < 0.001. JG19: Jigu 19; XJHG: Xiaojiahangu; JG31: Jigu 31; JG39: Jigu 39; YG1: Yugu 1. P: *P. oryzae*; P + B9: *P. oryzae* + NR2065B9.

The foxtail millet leaves of all six cultivars were drop-inoculated with conidia suspensions of DB165 on wounded sites, and the NR2065B9-treated group was conducted using strain NR2065B9 cell suspension on the same wound site. The results showed that the pathogen-only control group developed consistent and typical foxtail millet blast symptoms, with expanding necrotic lesions radiating outward from the inoculation sites along the leaf vein tissue (Figure 4). The final severity of blast symptoms was dependent on the host resistance background of each cultivar. On the resistant foxtail millet cultivar Xiaojiahangu (XJHG), lesions remained as tiny pinpoint necrotic spots strictly confined to the original inoculation sites. In contrast, on the susceptible standard control cultivar Ci846, expanding lesions formed at nearly all inoculation sites, resulting in significantly larger average lesion area than those recorded on XJHG. In the NR2065B9-treated group, disease incidence was markedly reduced across all six tested cultivars. Regardless of original host resistance level, lesions on treated leaves remained as small, localized necrotic spots strictly restricted to the inoculation sites. Quantitative measurement further confirmed that the final lesion area in the NR2065B9-treated group was significantly smaller than that of the corresponding pathogen-only control for every cultivar (Supplementary Table S9).

### 2.5. Biocontrol Efficacy of Antagonistic Bacteria NR2065B9 on Foxtail Millet Blast in Field Study

**Figure 5:**
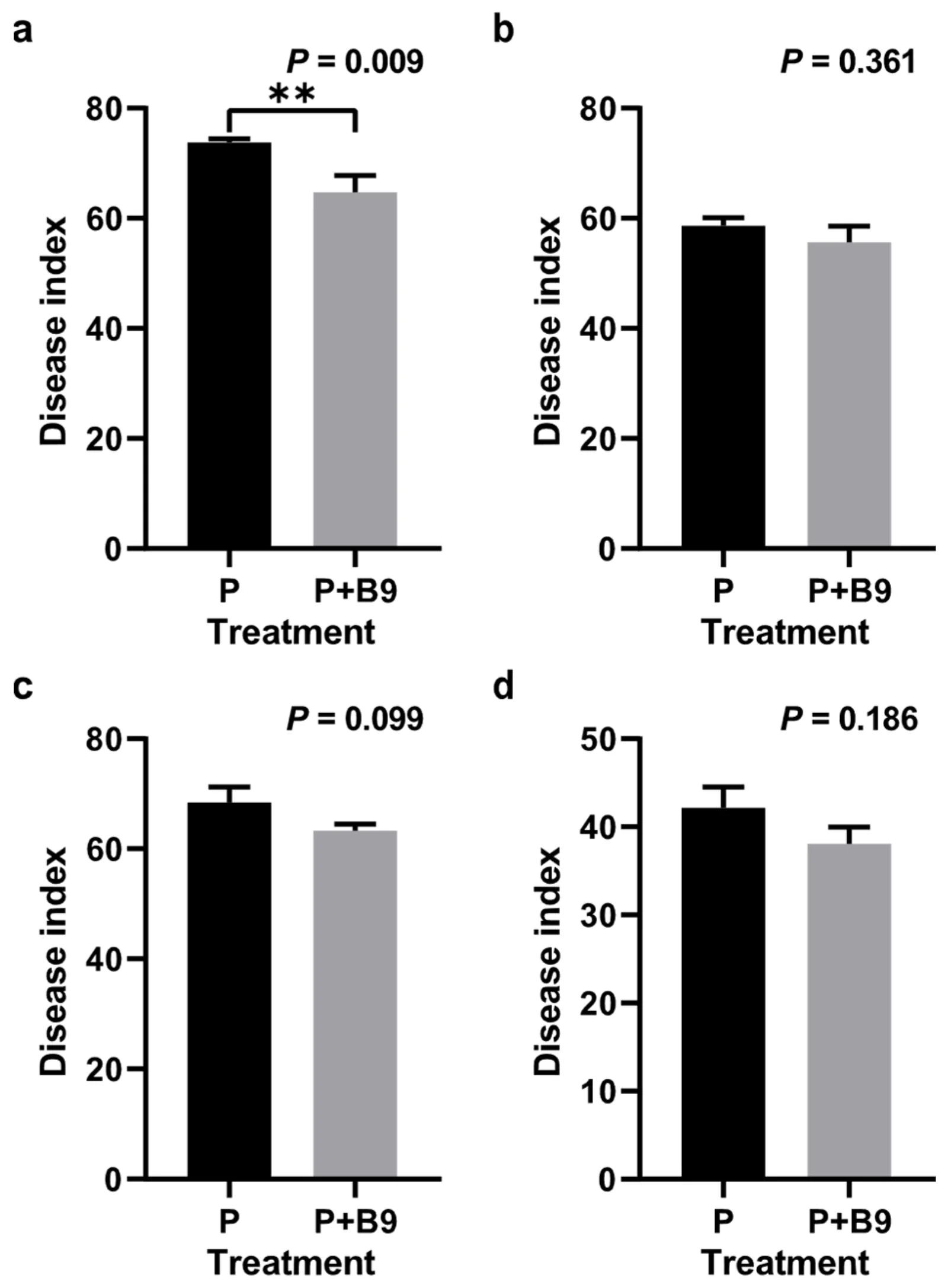
Effect of antagonistic bacterium *Pseudomonas silesiensis* NR2065B9 on foxtail millet blast control in four cultivars under field conditions. ** indicates *p* < 0.01. (**a**) Jigu 19. (**b**) Jigu 38. (**c**) Jigu 39. (**d**) Jichuanggu 1. P: *P. oryzae*; P + B9: *P. oryzae* + NR2065B9.

Strain NR2065B9 suppressed the disease severity of foxtail millet blast across four tested cultivars, namely JG19, JG38, JG39, and JCG1, under field inoculation conditions (Figure 5). Compared with the pathogen-only control treatment, application of NR2065B9 cell suspension resulted in a minimum disease control efficacy of 4.02% against foxtail millet blast across all cultivars (Supplementary Tables S10–S14). In contrast, plants in the control group, which were inoculated exclusively with *P. oryzae*, developed typical blast lesions and symptoms on all four cultivars under artificial inoculation.

## 3. Discussion

Foxtail millet is one of the most critical cereal crops in northern China [16]. In recent years, the large-scale intensive monocropping systems adopted for foxtail millet cultivation have aggravated the prevalence of airborne foliar and panicle diseases, with recurrent, severe outbreaks of foxtail millet blast caused by *P. oryzae* emerging as a major bottleneck that severely restricts the long-term sustainable development of the foxtail millet industry in China [8].

The dynamic interplay between bacterial and fungal communities in rhizosphere soil functions as the frontline defensive barrier for host plants, maintaining native microbial homeostasis, enhancing plant stress tolerance, and consequently bolstering host performance against a broad spectrum of biotic and abiotic stresses [17]. A previous research has demonstrated that upon exposure to the fungal root pathogen, multiple bacterial genera that natively colonize the sugar beet rhizosphere, notably *Paraburkholderia*, *Pseudomonas*, *Streptomyces*, *Chitinophaga* and *Flavobacterium*, act as a conserved defensive consortium to effectively suppress root disease progression [18]. Modulations in rhizosphere microbiome composition are widely documented to directly enhance host plant stress resilience. For instance, *Bacillus* species constitute the core microbiota actively recruited by maize cultivars resistant to stalk rot, to antagonize the phytopathogen [19]. Exogenous amendment of rhizosphere-associated bacteria affiliated with *Pseudomonas* and *Burkholderia* has also been proven to alleviate tobacco continuous cropping problems and promote host plant growth [20]. Collectively, these findings systematically demonstrate the non-substitutable functional role of soil beneficial microorganisms in plant disease management, which can deliver robust protection for healthy plant growth by blocking initial pathogen invasion. Targeted isolation and screening of functional strains with high biocontrol potential from foxtail millet rhizosphere soil is of great practical significance to advance sustainable green control strategies for foxtail millet diseases. At present, synthetic fungicide application remains the dominant strategy for crop disease management [21]. However, the widespread, large-scale and long-term overuse of chemical agents has triggered a series of adverse consequences, including severe disruption of the native agroecological environment, potential threats to human food safety, and the evolution of multi-fungicide resistance in pathogen populations [22]. The expanding adoption of biological control represents a highly viable alternative to reduce chemical input in sustainable agricultural systems. Accordingly, continuous exploration of elite, host-adapted functional bacterial strains is a core research priority for advancing modern biocontrol development, which aligns perfectly with the future development trend of green prevention and control technologies for foxtail millet diseases.

In the present study, a functional bacterial strain, designated NR2065B9, was isolated from the rhizosphere soil of healthy foxtail millet plants. In dual culture assays against the target fungal pathogen DB165, strain NR2065B9 inhibited the mycelial growth of *P. oryzae* by 98.77%, identifying it as a highly promising candidate both for mechanistic investigation and for biocontrol agent development. On the basis of morphological characterization combined with 16S rRNA gene phylogenetic analysis, the strain was taxonomically assigned to *P. silesiensis*. Members of *P. silesiensis* have been widely reported to perform diverse ecological functions, which fall into two broad categories. On the one hand, the species has repeatedly been shown to promote plant growth and to act as an effective biocontrol agent. The representatives of the species have improved the growth of maize in saline soil by modulating the structure and functional profile of the rhizosphere bacterial community [23], exerted pronounced plant growth-promoting effects when developed as a potential organic biofertilizer [24], and enhanced both host plant growth and tissue potassium acquisition and absorption through potassium solubilization [25]. On the other hand, *P. silesiensis* is extensively documented in pollutant degradation and environmental bioremediation: members of the species have efficiently biodegraded polyethylene [26], degraded phenanthrene and thereby improved bioremediation efficiency at contaminated sites [27], shown considerable potential for the bioremediation of pesticide-contaminated soil together with the sustainable production of biodegradable polyhydroxy acids or natural biosurfactants [28], and efficiently degraded the sulfonamide antibiotic sulfamethoxazole [29]. Considered together, these findings indicate that *P. silesiensis* possesses versatile application prospects in both plant growth promotion and environmental remediation. However, the antagonism towards phytopathogenic fungi has not previously been reported for this species. The strong antagonistic activity observed here against *P. oryzae* therefore represents a functional trait that is distinct from those described for other members of *P. silesiensis*. The present study identifies *P. silesiensis* NR2065B9 as a functional strain with antagonistic activity against the foxtail millet blast pathogen *P. oryzae*. This pronounced and previously unreported antagonistic performance merits further systematic exploration of the underlying biocontrol mechanisms and of the field application potential of this strain.

Strikingly, treatment with NR2065B9 drastically restricted pathogen colonization, resulting in only tiny, localized necrotic flecks confined strictly to the wound inoculation sites of foxtail millet leaves across six cultivars. This result indicated that strain NR2065B9 could effectively suppress *P. oryzae* lesion expansion on both resistant and susceptible foxtail millet genotypes, and its biocontrol performance remains stable across different host genetic backgrounds. The statistically significant reduction in lesion area for every cultivar demonstrated that the biocontrol activity of strain NR2065B9 was not genotype-dependent. This phenotype was consistent with previously reported modes of action for effective biocontrol agents [19]. The *Bacillus* biocontrol strain could recruit and induce berberine biosynthesis to achieve disease resistance across different maize genotypes, and its biocontrol activity was independent of the host-specific resistant germplasm. The observed genotype-independent biocontrol performance strongly indicated that strain NR2065B9 did not depend on variety-specific resistance pathways to exert its antifungal activity. It likely functioned either by directly suppressing the key early infection processes of *P. oryzae*, including appressorium formation and penetration, invasive hyphal differentiation, and subsequent necrotrophic phase expansion, or by triggering a genotype-nonspecific induced systemic resistance (ISR) pathway that was functional across diverse host germplasm [30,31,32,33,34]. Consistent with the observed indoor biocontrol performance, the field trial results demonstrated that foliar application of strain NR2065B9 could reduce blast disease severity in foxtail millet plants across all four tested cultivars, in contrast to the symptoms observed in the pathogen-inoculated control group. This field-suppressive effect further confirmed that strain NR2065B9 retained biocontrol activity under complex open-field conditions rather than being effective only in controlled indoor environments. However, the indoor dual culture and detached-leaf bioassays were conducted under strictly controlled laboratory conditions, where variables such as indigenous phyllosphere microbial competition, fluctuating field temperature and humidity, natural ultraviolet radiation, and mixed virulent pathogen populations were completely eliminated [35]. This highly stable, interference-free environment could fully release the direct antagonistic potential of strain NR2065B9, resulting in consistently high inhibition rates that were inherently higher than the performance achievable under open field conditions. Compared with the strictly controlled indoor assays, the actual field environment is far more complex: the indigenous soil microbial community, variable temperature and humidity fluctuations, natural pathogen population diversity, and the survival and colonization pressure of the applied biocontrol strain on the foxtail millet phyllosphere all together lead to the overall lower control efficacy than indoor results. This phenomenon is also consistent with the published field validation results of blast biocontrol agents, where most bacterial biocontrol strains show moderate field control effects significantly lower than their *in vitro* inhibition performance [7,36]. Subsequent field trials across multiple locations and growing seasons will be required to further validate the long-term stability of its biocontrol performance, optimize field application dosage and timing, and ultimately support the development of this strain into a commercially available bio-fungicide for sustainable foxtail millet blast management.

In addition, strain NR2065B9 exhibited significant inhibitory activity against the mycelial growth and vegetative hyphal expansion of the foxtail millet blast pathogen. A prior research has demonstrated a strong correlation between *in vitro* antagonistic performance and in planta pathogenicity suppression by *Pseudomonas* species in rice pathosystems [33]. The results of the present study supported that *in vitro* growth inhibition was correlated with *in vivo* disease suppression as observed during the dual culture assay and detached-leaf inoculation assay. Future work will dissect the unique antagonistic mechanism of strain NR2065B9 via activity-guided extraction and purification of its bioactive antimicrobial metabolites, to provide a theoretical foundation for the industrial development and large-scale field application of this antagonistic strain for sustainable foxtail millet blast management.

## 4. Materials and Methods

### 4.1. Foxtail Millet Soil Sampling

A total of twenty soil samples were collected from fields where foxtail millet blast occurred at the experimental station of the Institute of Millet Crops, Hebei Academy of Agriculture and Forestry Sciences (Supplementary Table S15). The plants with no disease characteristics were carefully uprooted and gently tapped to remove soil adhering to the roots, and the remaining soil tightly attached around the roots was considered as the rhizosphere soil. The collected rhizosphere soil was transported to the laboratory with dry ice and preserved at 4 °C for the isolation of bacterial cultivation.

### 4.2. Morphological and Cultural Characteristics and Identification of Foxtail Millet Blast Pathogen

A total of nine leaf samples with typical natural blast lesions were collected from symptomatic foxtail millet plants of three cultivars, including Jigu 19, Jingu 21 and Ci846 (Supplementary Table S16). Collected samples were stored at 4 °C for the isolation of pathogens. The pathogen was isolated using the method previously described [37]. The fungus was purified by a single spore isolation technique. A single foxtail millet blast pathogen isolate, designated DB165, was isolated from naturally infected foxtail millet blast leaf. This isolate was selected as the indicator pathogen for subsequent experiments, based on its stable and reproducible pathogenicity performance in repeated preliminary inoculation assays. Four-leaf-stage susceptible foxtail millet seedlings (cv. Ci846) were spray-inoculated with 1 × 10^5^ conidia/mL suspension of DB165. Typical blast lesions identical to field symptoms developed on all inoculated leaves after 3 days of incubation. The pathogen re-isolated from these lesions was identical to the original strain. The pathogenicity assay results were documented in Supplementary Figure S1.

The purified isolate DB165 was cultured on PDA medium. When the fungal colony had nearly fully covered the entire Petri dish, a sterile puncher was used to take mycelial plugs along the actively growing edge of the colony, which were then inoculated onto the center of fresh PDA and OTA plates at 26 °C for cultivation. Each sampling time point of colony growth was performed with five independent biological replicates, and the colony diameter of each plate was measured daily by cross-diameter measurement method. The morphology of colony and conidia was observed under an optical microscope and capture images using the cellSens software version 2.3 (Olympus, Japan).

The strain DB165 was inoculated on PDA medium, and the mycelia were harvested and immediately ground in liquid nitrogen for DNA extraction with cetyltrimethylammonium bromide (CTAB) buffer [38]. Equal volumes of supernatant and a mixture of phenol-chloroform-isoamyl alcohol was used to precipitate and isolate the DNA from other impurities. The purity and concentration of DNA was verified quantitatively and qualitatively using a NanoDrop spectrophotometer (Thermo Fisher Scientific, Waltham, MA, USA) and stored at −20 °C for later use.

The polymerase chain reaction (PCR) amplification was performed using a pair of universal primers ITS1 and ITS4 [39]. The PCR was performed in 20 μL reaction volume with 1 μL template DNA, 10 μL 2 × Taq PCR Master Mix, 0.5 μL forward primer, 0.5 μL reverse primer, and 8 μL sterile double-distilled water. The thermal cycling program was: initial denaturation at 95 °C for 5 min, followed by 35 cycles of denaturation at 95 °C for 15 s, annealing at 54 °C for 15 s, extension at 72 °C for 5 s, and final extension at 72 °C for 5 min. The amplified PCR products were verified by electrophoresis on 1% agarose gels, and then sequenced by Sangon Biotech (Shanghai, China). The DNA sequence was compared with the reference fungal species contained in a genomic database using the Basic Local Alignment Search Tool (BLAST (2026), https://blast.ncbi.nlm.nih.gov/Blast.cgi (7 September 2026)) from the National Center for Biotechnology Information (NCBI). The sequence similarity was calculated by the formula: (number of identical base pairs/total aligned base pairs) × 100%. Phylogenetic and molecular evolutionary analyses were conducted using MEGA software version 12 [40], with phylogenetic distances and clustering calculated using the Neighbor-Joining algorithm.

### 4.3. Isolation and Screening of Antagonistic Bacteria Against Foxtail Millet Blast Pathogen

The bacterial strains with antagonistic potential were isolated from foxtail millet rhizosphere soil [41]. A total of 10 g of soil was suspended in 100 mL of sterile distilled water and shaken at 28 °C and 180 rpm to liberate the microbes. The soil suspension was diluted by 10^6^-fold, and shortly after, 100 μL of diluent bacteria was evenly plated onto the LB solid medium. Through incubation at 28 °C, the individual colonies according to different morphologies were selected and successively streaked three times for further purification.

The isolated strains were individually incubated at 28 °C and 180 rpm for 24 h. Following incubation, the culture solutions were subjected to centrifugation at 5000 rpm for 10 min, followed by resuspension with sterile distilled water. Subsequently, the cell density was adjusted to an optical density at 600 nm (OD_600_) of 1.0, equivalent to a colony count of 1 × 10^7^ colony forming unit (CFU)/mL. A single streak of the bacterial suspension was inoculated onto one side of the PDA medium, while a mycelial plug of strain DB165 was placed on the opposite side of the medium, oriented perpendicularly to the inoculated bacterial streak. The PDA medium containing only DB165 was used as the non-treated control. The dual-culture assay was incubated at 26 °C, with five independent biological replicates set for each treatment. When the fungal hyphae of the control group had nearly fully covered the entire Petri dish, the cross-diameter measurement method was used to measure the colony diameter of the pathogenic fungus. The strains with significant antibacterial effects were selected and maintained in glycerol at −80 °C for long-term storage.

### 4.4. Identification of Antagonistic Bacteria Against Foxtail Millet Blast Pathogen

A total of nine bacterial strains with distinct antagonistic activity against *P. oryzae* were isolated from the collected field samples (Supplementary Table S5). One isolate (i.e., NR2065B9) was selected for further study based on its optimal plate confrontation inhibition performance. The isolated bacterial strain NR2065B9 was inoculated on LB solid medium using streaking method. The bacterial suspension was diluted with sterile distilled water. The diluent bacteria were evenly plated on the LB solid medium and incubated at 28 °C. Morphological characters including cell size and Gram staining of the isolated bacterial strain were observed under an optical microscope and capture images using the cellSens software. The Gram staining assay was performed following the classical four-step protocol [42]. A thin bacterial smear of the 24 h cultured strain was prepared on a clean glass slide, air-dried and fixed by gentle flaming. The smear was first stained with crystal violet for 1 min, then rinsed with sterile distilled water. After that, Gram’s iodine solution was added for mordant staining for 1 min, followed by washing. The slide was decolorized with 95% ethanol for 20–30 s until no purple dye eluted, then immediately rinsed with water. Finally, the smear was counterstained with safranin solution for 1 min, washed, air-dried and observed under an oil immersion microscope.

Identification of purified bacteria NR2065B9 was based on 16S rRNA gene sequences with universal forward primer 27F and reverse primer 1492R [43]. The PCR was performed in 20 μL reaction volume with 1 μL template DNA, 10 μL 2 × Taq PCR Master Mix, 0.5 μL forward primer, 0.5 µL reverse primer, and 8 µL sterile double-distilled water. The thermal cycling program was: initial denaturation at 95 °C for 3 min, followed by 35 cycles of denaturation at 95 °C for 15 s, annealing at 57 °C for 15 s, extension at 72 °C for 5 s, and final extension at 72 °C for 5 min. The amplified PCR products were verified by electrophoresis on 1% agarose gels, and then sequenced. The DNA sequence was compared with the reference bacterial species contained in a genomic database using the BLAST from the NCBI. Sequence alignment and phylogenetic tree construction were carried out using MEGA software, with phylogenetic distances and clustering calculated using the Neighbor-Joining algorithm.

### 4.5. In Vitro Antagonistic Activity of Functional Bacteria Against Foxtail Millet Blast Pathogen

Two complementary antagonism assays were performed to evaluate the inhibitory activity of the antagonistic strain NR2065B9 against target phytopathogen DB165. The strain NR2065B9 was inoculated in LB liquid medium and shaken at 28 °C and 180 rpm to obtain a bacterial suspension of the final concentration of 1 × 10^7^ CFU/mL. The fungal pathogenic isolate *P. oryzae* DB165 was cultured on PDA medium at 26 °C to obtain mycelial plugs for the *in vitro* antagonism tests.

The dual culture assay was conducted as method previously described [44], a 5 mm-diameter mycelial plug of the pathogen DB165 was inoculated at the center of PDA plate. The sterile paper discs which were impregnated with antagonistic bacterial cell suspensions were placed on the top, bottom, right, and left sides of the plate 20 mm away from the center of the plate. The PDA medium containing only pathogenic fungus was used as the non-treated control. Each dual culture antagonism assay was performed with five independent biological replicates. When the fungal hyphae of the control group had nearly fully covered the entire Petri dish, the irregular colony area of each treatment group was quantified using the irregular graph area calculation method previously described [45]. The formula for inhibition rate of the antagonistic bacteria against the foxtail millet blast pathogen was calculated according to the formula: inhibition rate (%) = ((control colony area − treatment colony area)/control colony area) × 100%.

For the mixed-medium plate assay, the antagonistic bacterial suspension was pre-mixed with molten PDA medium at a final concentration of 10^7^ CFU/mL, then poured into sterile Petri dishes. After solidification, a 5 mm pathogen mycelial plug was inoculated at the plate center. Each treatment was performed with five independent biological replicates. The plates were incubated at 26 °C for 7 d. The colony area of the pathogen DB165 was recorded, and the inhibition rate was calculated using the untreated PDA plates without bacterial addition as the control.

### 4.6. Suppressive Effect of Antagonistic Bacteria on Symptom Expression of Foxtail Millet Blast in Detached-Leaf Inoculation Assay

Six foxtail millet cultivars, namely Ci846, Jigu 19 (JG19), Xiaojiahangu (XJHG), Jigu 31 (JG31), Jigu 39 (JG39), and Yugu 1 (YG1), with differential millet blast resistance, including both susceptible and resistant genotypes were used for indoor detached-leaf inoculation assay. The blast-susceptible cultivar Ci846 was designated as the standard susceptible control. For each tested foxtail millet cultivar, 6 uniform and healthy seedlings were retained per pot for subsequent inoculation treatment. The plants were grown in a greenhouse at 26 to 32 °C with a light and dark cycle of 16 and 8 h.

The pathogen *P. oryzae* DB165 was cultured on OTA medium at 26 °C for 7 d to produce conidia [46]. The conidia were collected through adding 2 mL of sterilized water to OTA plate and gently scraping the entire colony surface using a sterilized glass spreader [47]. The conidial suspension was filtered through three layers of sterile gauze to remove residual mycelial debris and medium fragments. The initial concentration was roughly estimated under an optical microscope with a hemocytometer, and then subjected to 10-fold serial gradient dilution with sterile water. The final concentration of the conidial suspension was adjusted precisely to 1 × 10^5^ spores/mL prior to its application for subsequent inoculation tests.

At the 6-leaf growth stage of foxtail millet, the penultimate leaf on each plant was collected, and cut into 8 cm-long segments. Using a sterile needle to scrape 3 wounds in the main veins of detached leaves. Each leaf segment was inoculated with three droplets of *P. oryzae* DB165 conidial suspension. Each treatment was performed with nine independent biological replicates, with 3 individual wound sites on each leaf defined as one independent replicate unit. For the NR2065B9-treated group, an equal volume of NR2065B9 cell suspension was additionally applied to the same wound site after pathogen inoculation, while the pathogen-only control group received an equivalent volume of sterile water instead of the antagonistic bacterial suspension. Segments of detached leaves were placed onto Petri dishes containing 6-benzylaminopurine aqueous solution. The Petri dishes were placed in an incubator in darkness at 26 °C for 24 h, and subsequently kept under a 12/12 (day/night) photoperiod. After 3 days of inoculation, the disease severity of foxtail millet leaves was assessed according to the previous study [48]. The size of leaf lesions was measured using ImageJ software version 1.53e [49].

### 4.7. Field Trial Design and Assessment of Foxtail Millet Blast Suppression by Antagonistic Strain

The field trial was conducted in order to evaluate the suppressive effect of inoculating strain NR2065B9 on field-grown foxtail millet plants. Four dominant cultivars currently widely planted in the core foxtail millet production regions of Hebei Province were deliberately selected: Jigu 19 (JG19), Jigu 38 (JG38), Jigu 39 (JG39), and Jichuanggu 1 (JCG1). The experimental setup consisted of two distinct treatments: a non-antagonist-inoculated control, and a NR2065B9 bacterial inoculation treatment.

When all plants reached the uniform 6-leaf growth stage, two groups of treatments were set up for the foxtail millet blast biocontrol assay. All test plants were first uniformly spray-inoculated with the *P. oryzae* conidial suspension at a concentration of 1 × 10^5^ spores/mL. After that, the NR2065B9 treatment group was foliar-sprayed with the antagonistic bacterial suspension at a concentration of 1 × 10^7^ CFU/mL, while the blank control group was sprayed with equal volume of sterile water. Two days after the initial spray inoculation, each individual plants in the NR2065B9 treatment group received a second inoculation of bacterial suspension. Each treatment was composed of six independent 2 m-long planting rows as biological replicates arranged for every foxtail millet cultivar. Approximately 30 plants were retained in each row after thinning.

Four weeks after the second antagonistic bacterial inoculation, the foxtail millet blast disease severity of plants was surveyed. For each treatment, 20 uniform plants with consistent growth status were randomly selected, and each individual plant was evaluated using the 1–9 scale as previously documented with minor modifications [50]. The collected disease grades were subsequently converted into the disease index (DI) using the validated formula [51]:

DI = ∑ (All individual severity ratings)/(Total number of observed leaves × Maximum disease grade value) × 100

The relative biocontrol efficacy of strain NR2065B9 against foxtail millet blast was finally calculated with the following formula:

Disease control efficacy (%) = [ (DI of control group − DI of treatment group)/DI of control group] × 100

### 4.8. Statistical Analyses

All data, including mycelial inhibition rate, leaf lesion size, and field disease index of foxtail millet blast, were subjected to independent samples Student’s *t*-test for comparison between different treatments. The significance threshold was set at *p* < 0.05, and all statistical analyses were executed on IBM SPSS Statistics software version 21. Bar charts for intuitive visualization of results were generated using GraphPad Prism software version 8 (Boston, MA, USA).

## 5. Conclusions

In this study, a bacterial strain *P. silesiensis* NR2065B9 was isolated from the rhizosphere soil of healthy foxtail millet plants. This strain exhibited significant antagonistic activity against the causal agent of foxtail millet blast *P. oryzae*, with a mycelial growth inhibition rate reaching 98.77%. Consistent with the *in vitro* performance, the field test results demonstrated that the application of strain NR2065B9 suspension was able to reduce foxtail millet blast severity in all tested inoculated cultivars. The verified *in vitro* and field antagonistic performance of strain NR2065B9 provides a valuable native bacterial resource for the biocontrol of foxtail millet blast, and indicates that this strain has promising potential to be developed as an efficient biocontrol candidate for foxtail millet disease management in sustainable agricultural production. Further research will be carried out to clarify the specific antagonistic mechanism of strain NR2065B9 via transcriptomics and metabolomics integrated multi-omics approach, and subsequent multi-location consecutive field trials will be conducted to systematically validate its practical field control efficacy against foxtail millet blast.

## Data Availability

All data are displayed in the manuscript and Supplementary Files.

## Supplementary Materials

The following supporting information can be downloaded at https://www.mdpi.com/article/10.3390/plants15213289/s1, Figure S1. Pathogenicity verification of *Pyricularia oryzae* strain DB165 on foxtail millet. Table S1. The conidium size of strain DB165; Table S2. The diameter of strain DB165 on the PDA medium; Table S3. The diameter of strain DB165 on the OTA medium; Table S4. The alignment results and reference isolate information for phylogenetic identification of the two target strains; Table S5. The pathogen colony area and mycelial growth inhibition rates of 9 isolates under different treatments; Table S6. The cell size of bacterial strain NR2065B9; Table S7. The pathogen colony area and mycelial growth inhibition rates of treatments with or without strain NR2065B9; Table S8. The pathogen colony area and mycelial growth inhibition rates of two agar media; Table S9. Leaf lesion area of treatments with or without strain NR2065B9; Table S10. Disease grade of Jigu 19 of treatments with or without strain NR2065B9; Table S11. Disease grade of Jigu 38 of treatments with or without strain NR2065B9; Table S12. Disease grade of Jigu 39 of treatments with or without strain NR2065B9; Table S13. Disease grade of Jichuanggu 1 of treatments with or without strain NR2065B9; Table S14. Disease control efficacies of antagonistic bacterium NR2065B9 on foxtail millet blast in four cultivars under field conditions; Table S15. Information of soil samples in foxtail millet field; Table S16. Information of leaf samples in foxtail millet field.
