# Supplementary material for: Isolation, Identification and Antifungal Activity Determination of Antagonistic Bacteria Against Pyricularia oryzae Causing Foxtail Millet Blast
Source: Plants (Basel). 2026 Oct 25;15(21):3289. doi: 10.3390/plants15213289 (PMC13765671; doi:10.3390/plants15213289)
Supplement: Supplementary file 1 [file plants-15-03289-s001.zip › Supplementary Figures 2026.10.12.pdf]

a b
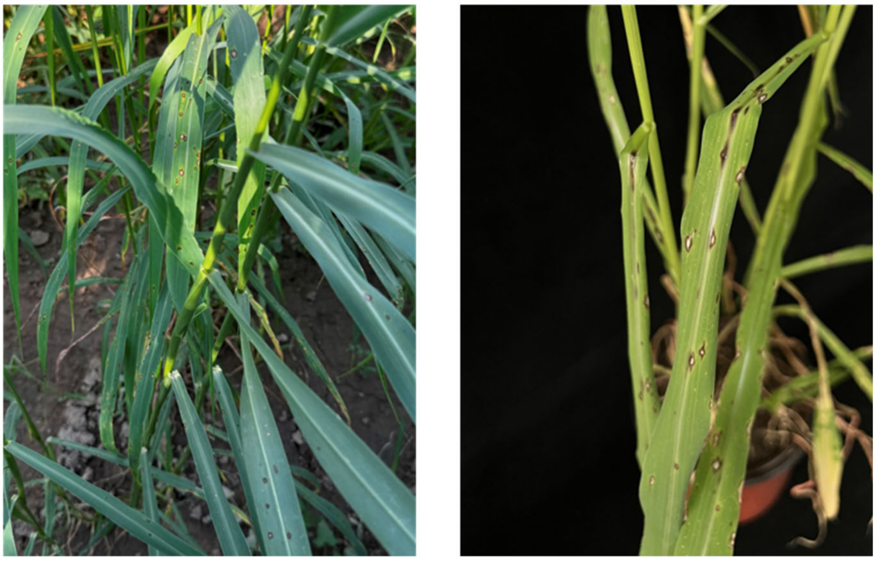

Figure S1. Pathogenicity verification of *Pyricularia oryzae* strain DB165 on foxtail millet. (**a**) Typical natural blast lesions on field-collected symptomatic foxtail millet leaves. (**b**) Reproduced typical blast lesions on healthy susceptible foxtail millet leaves 3 days post indoor inoculation with the purified single-spore isolate DB165.
